# Effects of Health Service Utilization and Informal Social Support on Depression, Anxiety, and Stress among the Internal Migrant Elderly following Children in Weifang, China

**DOI:** 10.3390/ijerph192214640

**Published:** 2022-11-08

**Authors:** Hexian Li, Mingli Pang, Jieru Wang, Jing Xu, Fanlei Kong

**Affiliations:** 1 Centre for Health Management and Policy Research, School of Public Health, Cheeloo College of Medicine, Shandong University, Jinan 250012, China; 2 NHC Key Lab of Health Economics and Policy Research, Shandong University, Jinan 250012, China

**Keywords:** internal migrant elderly following children, depression, anxiety, stress, informal social support

## Abstract

This study explored the relationship between health service utilization, informal social support and depression, anxiety and stress among the internal migrant elderly following children (IMEFC) in Weifang, China. A total of 613 IMEFC were selected using multistage cluster random sampling. The Depression Anxiety and Stress Scale 21 (DASS-21) was used to assess the depression, anxiety and stress of the IMEFC. Descriptive analysis and univariate and binary logistic regression analyses were used to clarify the correlation between health service utilization and social support and depression, anxiety and stress of the IMEFC. The prevalence of depression, anxiety and stress of the IMEFC was 6.9%, 7.7% and 3.4%, respectively. Logistic regression analysis showed that the IMEFC who having financial stress on medical costs were more likely to feel depressed than those haven’t financial stress on medical costs (OR = 6.557), while those unemployed and having no income were less likely to feel depressed than those employed (OR = 0.262), having children support were less likely to feel depressed than those haven’t children support (OR = 0.257) and having comfort support were less likely to feel depressed than haven’t comfort support (OR = 0.018). Trans-city migration were more likely to feel anxious than trans-county migration (OR = 3.198), having outpatient service were more likely to feel anxious than haven’t experienced inpatient service (OR = 3.818), having financial stress on medical costs were more likely to feel anxious than haven’t financial stress on medical costs (OR = 3.726), while having children support were less likely to feel anxious than haven’t children support (OR = 0.198). Those who migrate to cure disease or rehabilitation were more likely to feel stressed than those migrated to taking care of grandchildren (OR = 12.702) and having financial stress on medical costs were more likely to feel stressed than haven’t financial stress on medical costs (OR = 32.155), while having children support were less likely to feel stressed than haven’t children support (OR = 0.055) and having economic support in troubles were less likely to feel stressed than haven’t economic support in troubles (OR = 0.012). More effective measures should be taken to improve the accessibility and efficiency of cross-regional health insurance reimbursement, and family members should spend more time with the IMEFC to lower their psychological tension in a new environment.

## 1. Introduction

The number and proportion of older adults have been increasing in the past decades and have become one of the global demographic challenges [1]. As the largest developing country, China’s aging population has also increased rapidly. At the end of 2021, its citizens aged 65 and above were 20.56 million, accounting for 14.2% of the total population, up 0.7% from 2020 [2]. The United Nations projects that by 2030 adults over 65 years old will represent approximately 16.9% of the country’s population [3]. As its people age and urbanization accelerates, many young persons are leaving their hometowns and choosing to settle in big cities. By 2020, China’s migrants were 375.82 million, 69.73% more than in 2010 [4], and 124.84 million were cross-provincial ones. In this context, a large number of the elderly joined the migration to reunite with their families and take care of their grandchildren. In 2015, the number of elderly migrants reached 13.04 million, accounting for 5.3% of China’s whole migrant population [5]. In this study, those who leave the familiar rural or urban environment and follow their children to big cities to take care of their grandchild or being taken care of by their children are referred to as the internal migrant elderly following children (IMEFC) [6].

Migration can be a very stress-inducing phenomenon [7]. Previous research has shown that the migration experience has a significant impact on mental health, raising the risk of developing depression and anxiety [8]. The *hukou* system, one of the main administrative systems in the country, is a major barrier to socioeconomic mobility and may exacerbate health inequalities in China [9]. Older migrant adults are often not entitled to the same social benefits and health services as local residents and are therefore more likely to face physical and mental health problems [10,11,12]. Low socioeconomic status, combined with limited social benefits, has led to lower healthcare utilization. Often, migrant older adults prefer to receive self-medication or no treatment rather than go to the hospital [13]. Several studies have shown that migration may affect the migrants’ mental health because of the decline in physical fitness [9,14]. The existing literature showed the prevalence of anxiety and depression among elderly migrants has increased and become an important issue in China [15,16,17], including for the IMEFC [18]. The mental health of the migrant elderly was more affected than that of the urban elderly, with depression found to be more prevalent among them [19]. According to a study on elderly migrants in Hangzhou, 35.9% of them had a tendency to be depressed, which was a significantly higher rate than that of the general elderly population. Living arrangements, social support and physical health status are important influencing factors [20]. Therefore, more attention should be given to the mental health of the IMEFC.

Social support is understood as the subjective experience that a person is loved, cared for, respected and valued by others, who could be partners, relatives, friends, co-workers, society and community members [21]. It can be divided into formal and informal social support [22]. According to existing research on older adults in China, organizations at all levels of the government, institutions, businesses and communities are considered part of formal support while family members, neighbors, friends and peers supply informal support [23]. Previous studies have confirmed the significantly positive impact of social support on mental health [24,25]. Conversely, social support is negatively correlated with depression, which it can actually alleviate [26,27]. A study in China showed that the IMEFC received less social support and had a lower sense of happiness than their local counterparts [28,29]. Strengthening social support for elderly migrants was also concluded to be necessary to promote their adaptation and lower their stress [30]. A study on older adults over 60 in Hong Kong found that informal support from family members, relatives, friends or neighbors was effective in enhancing their mental health [31]. Another existed study also found that informal support, such as financial support and companionship, had a significant positive impact on older adults’ health [32]. As most IMEFC are from rural areas, they may rely more on informal social support as they receive inadequate formal social support in areas such as healthcare and public services [33]. The effect of informal social support on the mental health of the IMEFC deserves more attention.

Given the above background, there is no study that simultaneously explored the effects of health service utilization and informal social support on depression, anxiety and stress of IMEFC, let alone takes IMEFC as the research object. To fill this research gap, this study aimed to clarify the effects of health service utilization and informal social support on depression, anxiety and stress of IMEFC in Weifang, China.

## 2. Materials and Methods

### 2.1. Data Collection and Research Subjects

The data were collected in the city of Weifang, Shandong Province, China, in August 2021. The study included people 60 years and older who followed their children to live in Weifang county. Multi-stage cluster random sampling was used to select the participants. In the first stage of the data collection, the 4 most developed districts were chosen from the 12 districts as the primary sampling units (PSUs), considering their economic development and geographic location. In the second stage, a total of four sub-districts were selected from each of the PSUs as the secondary sampling units (SSUs); that is, one sub-district was selected from each of the districts chosen previously. In the third stage, four communities were selected from each of the SSUs; that is, one community was selected from each of the sub-districts chosen previously. All the migrants who were older than 60 years old and who had followed their children to Weifang in the above communities constituted the total sample of this study. The inclusion criteria for participants were (1) aged 60 or older, (2) non-local residents in the household registration area, and (3) able to understand Mandarin and communicate smoothly with the surveyors.

In total, 25 university students became investigators for this study after receiving training regarding the background information of the study, contents of the questionnaire and techniques of a social survey. The investigators conducted approximately 20 min face-to-face interviews with the subjects to collect the data. A total of 616 IMEFC were initially selected and interviewed. However, 3 of them were excluded due to the logical errors in their responses or uncompleted questionnaires. A total of 613 participants were ultimately included in the study.

### 2.2. Measurements

#### 2.2.1. Depression, Anxiety and Stress

The Depression, Anxiety and Stress Scale 21 (DASS-21) was adopted to measure mental health in participants, who were required to answer seven items for each dimension (depression, anxiety and stress). Questions 3, 5, 10, 13, 16, 17 and 21 constitute the depression subscale. Questions 2, 4, 7, 9, 15, 19 and 20 form the anxiety subscale, and questions 1, 6, 8, 11, 12, 14 and 18 form the stress scale. Responses ranged from 0 to 3, with 0 indicating “did not apply to me at all”, 1 meaning “applied to me to some degree, or some of the time”, 2 standing for “applied to me to a considerable degree or a good part of time” and 3 for “applied to me very much or most of the time” [34]. The DASS-21 is a short version of the original 42-item DASS form; therefore, to assess the level of severity among the target population, the DASS-21 score was multiplied by two. The severity of depression was classified into five ranges, normal (0–9), mild (10–13), moderate (14–20), severe (21–27) and very severe (28+). Similarly, the anxiety-level ranges were normal (0–7), mild (8–9), moderate (10–14), severe (15–19) and very severe (20+). Stress scores ranged from normal 0–14, to mild 15–18, moderate 19–25, severe 26–33 and extremely severe 34+. Similar to a previous study, scores obtained on these three subscales were dichotomized [35]. Those falling in moderately, severely and extremely severely depressed, anxious or stressed categories were considered depressed (total score > 9), anxious (total score > 7) and stressed (total score > 14), respectively. Others were considered not depressed (total score ≤ 9), not anxious (total score ≤ 7) and not stressed (total score ≤ 14). DASS-21 has been proven to be a reliable and effective measure to assess the mental health of the Chinese population including older adults [36,37].

#### 2.2.2. Social Demographic Characteristics

Social demographic characteristics, collected as a basis for comparison, included age, sex, marital status, education, *hukou* (one of China’s oldest tools for population control, which is essentially a household registration permit, akin to an internal passport [38]), monthly income, employment, migration reason, migration range and hesitancy to migrate.

#### 2.2.3. Health Service Utilization

Health service utilization was captured by seven indicators, including chronic diseases (no, yes), health status (good, poor), outpatient service experience in past year (no, yes), inpatient service experience in past year (no, yes), having ever gone to a hospital alone for fear of bothering their children (no, yes), having ever purchased medicine alone for fear of bothering their children (no, yes) and financial stress on medical costs (no, moderate, yes).

#### 2.2.4. Informal Social Support

Social support can be divided into formal social support and informal social support. Formal social support includes formal organizations at all levels of government, institutions, businesses and communities. Informal social support usually comes from family members, neighbors, friends and peers [23]. Based on this, this study used eight questions to measure informal social support: relationships with neighbors (bad, fairly bad, fairly good, good), relationships with friends (bad, fairly bad, fairly good, good), couple support (no, yes), children’s support (no, yes), siblings’ support (no, yes), other members’ support (no, yes), having ever received the economic support when in trouble (no, yes) and having ever received comfort support when in trouble (no, yes).

#### 2.2.5. Statistical Analysis

All statistical analyses were performed using SPSS26.0 (International Business Machines Corporation, Armonk, NY, USA), and *p*-values less than 0.05 were regarded as statistically significant [39]. Chi-square test and Fisher exact test were used to calculate the differences in depression, anxiety and stress among the subgroups of each categorical variable. After univariate analyses, statistically significant variables were included in the logistic regression analyses. Three binary logistic regression models with an enter method were adopted to explore the associations between independent variables and depression, anxiety and stress. Meanwhile, crude odds ratios (OR) and 95% confidence intervals (95% CI) were calculated. In Model 1, the independent variables included the basic demographic characteristic variables, Model 2 added health service utilization-related variables to Model 1, while Model 3 added social support-related variables to Model 2.

## 3. Results

### 3.1. Participant Characteristics

Table 1 provides the basic information on social demographic characteristics, health service utilization, informal social support for 613 participants, of which 55.8% were 60 to 65 years old. More than half of the participants were female (73.1%), married (87.9%) and educated below upper-secondary level (82.2%). Most of the IMEFC (83.4%) had a monthly income level of below CNY 2000. Meanwhile, 85.6% of them were rural *hukou,* and 70.8% were unemployed. Furthermore, the majority had migrated to take care of their grandchildren (86.9%), mostly to another county (70.1%). More than half (83.5%) were not at all hesitant about migration. According to the results of the Chi-square test, gender (*p* = 0.016), age (*p* = 0.038), employment (*p* = 0.040) and migration reason (*p* = 0.028) were significantly associated with depression among IMEFC. Factors that were significantly different in relation to anxiety included marital status (*p* = 0.013), migration reason (*p* = 0.01) and migration range (*p* = 0.002). Concerning stress, there were statistically significant differences in employment (*p* = 0.018), migration reason (*p* = 0.016) and hesitancy to migrate (*p* = 0.019).

### 3.2. Health Service Utilization

As shown in Table 1, 42.7% of respondents had chronic diseases, and 25.8% were in moderate or poor health. Most of the IMEFC had had no outpatient service experience in the past year (72.6%), while 83.0% had had no inpatient service experience during the same period of time. A total of 54.0% of the respondents had never gone to a hospital alone, and 44.9% of them had not bought medicine alone. Most of them stated that they were financially stressed about medical costs (62%). Chi-square test illustrated that chronic diseases, health status, outpatient service utilization, inpatient service experience, going to a hospital alone and stress about medical cost were significantly associated with depression, anxiety and stress.

### 3.3. Informal Social Support

The majority of the participants had good relationships with their neighbors (48.6%) and friends (48.1%). Most of the IMEFC stated that they were receiving support from their partner (83.7%), children (94.1%), siblings (51.5%) and other family members (50.7%), as well as economic (97.6%) and comfort support when in trouble (99.0%). The Chi-square test analysis results showed that factors including relationships with neighbors (*p* = 0.001) and friends (*p* = 0.002), children’s support (*p* = 0.001), siblings’ (*p* = 0.038) and other members’ support (*p* = 0.014), economic support (*p* = 0.015) and comfort support when in trouble (*p* = 0.005) were statistically significantly associated with depression. The factors that were significantly different in relation to anxiety included relationships with neighbors (*p* = 0.006) and with friends (*p* = 0.008), couple support (*p* = 0.009), children’s support (*p* < 0.001) and siblings’ support (*p* = 0.012). Regarding stress, there were statistically significant differences in terms of children’s support (*p* = 0.001) and economic support when in trouble (*p* < 0.001).

### 3.4. The Association between Demographic Characteristics, Health Service Utilization, Informal Social Support and Depression

A logistic regression analysis was conducted to demonstrate the relationship between health service utilization, informal social support and depression (as illustrated in Table 2). In Model 1, basic demographic variables were included, and the results indicated that employment and migration reason were statistically significantly associated with depression. In Model 2, both basic demographic information and health service utilization were included. The results showed that the IMEFC who had inpatient service experiences were more likely to feel depressed than those who had not (OR = 3.496). Model 3 included demographics, health service utilization and informal social support variables. It was found that employment, having had inpatient service experiences, financial stress on medical costs, children’s support and comfort support when in trouble were all statistically significantly correlated with depression. In particular, the financially stressed IMEFC were more likely to feel depressed than those without such stress (OR = 6.557). Unemployed and no-income respondents were less likely to feel depressed than those who were employed (OR = 0.262). Those receiving children’s support were less likely to feeling depressed than those not benefitting from such support (OR = 0.257), and those having comfort support when in trouble were less likely to have depression than those not having support (OR = 0.018).

### 3.5. Association between Demographic Characteristics Health Service Utilization, Informal Social Support and Anxiety

As shown in Table 3, a logistic regression analysis was conducted to demonstrate the relationship between health service utilization, informal social support and anxiety. The results of Model 1 indicated that migration reason and migration range were statistically significantly associated with anxiety among IMEFC. In Model 2, the results showed that people who had inpatient service experiences in the past year and financial stress on medical costs were statistically significantly more inclined toward anxiety. Model 3 revealed that migration reason, migration range, inpatient service experiences, financial stress on medical costs and children’s support were statistically significantly correlated with anxiety. Specifically, the IMEFC who migrated to cure a disease or for rehabilitation purposes were more likely to feel anxious than those who had migrated to take care of grandchildren (OR = 3.837); trans-city migrants were more likely to feel anxious than trans-county ones (OR = 3.198); those being financially stressed about medical costs were more likely to feel anxious than those not being stressed (OR = 3.726). The IMEFC who benefitted from children’s support were less likely to feel anxious than those who did not (OR = 0.198).

### 3.6. Association between Demographic Characteristics, Health Service Utilization, Informal Social Support and Stress

Table 4 presents the results of the logistic regression analysis on the relationship between health service utilization, informal social support and stress. As seen, employment status, migration reason and hesitancy to migrate were statistically significantly associated with stress among IMEFC in Model 1. In Model 2, the results proved that financially stressed respondents were statistically significantly more affected by stress. Model 3 showed that demographic characteristics, health service utilization and informal social support were also statistically significantly associated with stress. Specifically, the IMEFC who had migrated to cure a disease or to rehabilitate were more likely to feel stressed than those who had migrated to take care of grandchildren (OR = 12.702), and those who had medical costs were more likely to feel stressed than those who had none (OR = 32.155). Inversely, those receiving children’s support were less likely to feel stressed than those deprived of such support (OR = 0.055), and having economic support when in trouble meant respondents were less likely to feel stressed than their peers without support (OR = 0.012).

## 4. Discussion

### 4.1. The Level of Depression, Anxiety and Stress among the IMEFC in Weifang, Shandong Province

In this study, the prevalence of depression, anxiety and stress among the IMEFC was 6.9%, 7.7% and 3.4%, respectively. These values were lower than the prevalence of depression and anxiety among the elderly migrants in Shandong Province, China [15]. The reason for this may be participants’ different characteristics between Yu’s and our study, as well as the use of different scales to measure the level of depression and anxiety, the incidence of depression among the elderly in urban Sichuan Province [40] and the incidence of depression and anxiety among the elderly in rural Guizhou Province, China [41]. Another reason for this lower prevalence could be the fact that most of our respondents were able to enjoy a higher standard of living and more time together with their family members. From a macro perspective, Confucianism culture is predominant in China, especially in Shandong Province [42]. It particularly emphasizes the importance of mutual support between family members, family unions and social harmony [43].Thus, living with their offspring would generally help the IMEFC enjoy their life and lower their depression, anxiety and stress levels. In addition, it is worth noting that the results of this study showed a higher incidence of depression than that observed among the general elderly population in Shanghai, China [44] and a higher incidence of anxiety than among the empty nesters in Anhui Province, China [45], indicating that the mental health of IMEFC still deserves more attention.

### 4.2. Association between Demographic Characteristics and Depression, Anxiety and Stress

This study found that employment status was statistically significantly associated with depression, which was similar with the results of previous studies. A Japanese study showed that unemployment was associated with migrants’ self-rated poor mental health [46]. A U.S. study reported that unemployment may have a negative impact on immigrants’ mental health [47]. However, this is contrary to our findings. The reason for those who were unemployed and had no income being less likely to feel depressed may be two-fold. Firstly, their family members might have had a high income or might have been wealthy enough to make it unnecessary for these IMEFC to work and allow them to just stay at home and take care of their grandchildren. Secondly, the situation might be the reverse, meaning that these IMEFC may have come from poor families and thus were easily satisfied and enjoyed family unions in the inflow cities. Conversely, those IMEFC who still needed to work may have felt the burden from both their jobs and caring for grandchildren and were thus more prone to be depressed.

Migration range was found to be statistically significantly correlated with anxiety; specifically, trans-city migrant IMEFC were more likely to be anxious than trans-county ones, which was consistent with previous research results. Migrant elders leave their homes and their familiar social and societal environment [48], migration over long distances takes a higher toll on them, and their daily lives change dramatically. All of these could affect their physical and mental health [49], including by causing anxiety.

Migration reason was observed to be statistically significantly associated with stress in this study. Specifically, the IMEFC who had migrated to cure a disease or rehabilitate were more likely to be stressed than those who migrated to care for their grandchildren. This may firstly be explained by the fact that most of the IMEFC’s children are aged 35–40, which is the starting stage of career development. As such, they greatly need support from their family members, friends and colleagues. However, those IMEFCs who followed their children to new cities to seek medical treatment will require more time and burden their children with some of their problems. This might eventually cause the IMEFC to feel regretful and stressed, and it is widely acknowledged that physical health is closely linked to mental health. As such, poor physical health may increase the risk of poor mental health [50] for the IMEFC.

### 4.3. Association between Health Service Utilization and Depression, Anxiety and Stress

The findings of this study showed that financial stress about healthcare costs was statistically significantly associated with anxiety, depression and stress, while utilizing inpatient services was also significantly associated with the depression and anxiety of IMEFC. As age increases, physical ability generally declines, and medical costs increase, affecting older adults’ mental health and leading to symptoms such as anxiety and depression [15,51]. Our results were consistent with those of previous research. The IMEFC are a unique group of migrants that formed during China’s urbanization process. Existing studies have found that the overall physical and mental health of elderly migrants is poorer than that of non-migrants [52]. Although the former joined their hometown’s social security and medical insurance system, they are often excluded from that of the inflow city. The current healthcare delivery system does not encourage them to seek appropriate health services; thus, they may be prone to higher financial stress and associated anxiety, depression and other forms of stress [52,53].

### 4.4. Association between Informal Social Support and Depression, Anxiety and Stress

Support from children was found to be significantly associated with anxiety, depression and stress among IMEFC. Similar to previous studies, our results showed that the degree of filial piety was positively correlated with the elderly’s mental health [54,55,56]. Moreover, Dong et al.’s study indicated older adults with higher-than-expected filial piety tended to be less vulnerable to depressive symptoms [57],

The IMEFC with no comfort support when in trouble were identified as more likely to feel depressed than those receiving such support in this study. This might be due to the fact that IMEFC fell out of touch with their acquaintances after migrating to new cities and were not able to find someone familiar to talk and share their problems. This might have eventually caused mental health problems [58].

There have been few studies examining the relationship between economic support and stress, as well as the elderly migrants. Thus, we expanded the target group to the general older adults. We found that economic support in trouble was statistically significantly associated with stress; particularly the IMEFC without economic support were more likely to be stressed than those who had this. As China is a country that deeply values filial piety [59], the older adults rely more on their children and relatives for financial support. The majority of the IMEFC in this study were unemployed and had no income; as such, they were mostly economically disadvantaged and more likely to be stressed from a lack of economic support.

### 4.5. Implications

First, the government could improve the accessibility and efficiency of cross-regional health insurances for IMEFC to reduce their medical cost pressure, with the help of the online national healthcare system and network technology. Second, communities could organize more recreational activities with the help of the social workers to increase opportunities for the IMEFC to establish close friendships and trust with locals. Third, IMEFC’s children should spend more time and energy with their parents to help reduce their psychological tensions in the new environment.

### 4.6. Limitations

First, eight questions from previous references and questionnaires were used to assess informal social support in this study since there is no existing scale to measure the informal social support. Second, this study had a cross-sectional design, so it could not determine the causal relationship between health service utilization and informal social support, on one side, and depression, anxiety and stress, on the other. Third, we used the DASS-21 scale to assess depression, anxiety and stress and did not use clinical diagnoses based on the ICD-11 and DSM-5. Finally, due to the COVID-19 pandemic, data collection was only finished in Weifang, while the questionnaire survey in Shanghai could not be completed as planned. This additional location may have provided a clearer picture on the IMEFC in China.

## 5. Conclusions

The prevalence of depression, anxiety and stress of IMEFC was 6.9%, 7.7% and 3.4%, respectively, in this study. Employment status, inpatient service experiences, financial stress over medical costs, children’s support and comfort support when in trouble were found to be statistically significantly associated with depression among IMEFC. Migration range, inpatient service experiences and children’s support were statistically significantly associated with anxiety in this population. Migration reason, financial stress on medical costs, children’s support and economic support when in trouble were statistically significantly associated with stress.

## Figures and Tables

**Table 1:** Descriptive analysis of the general characteristics of IMEFC by depression, anxiety and stress.

Variables	Total	Depression			Anxiety			Stress		
	N (%)	No *n* (%)	Yes *n* (%)	*p*	No *n* (%)	Yes *n* (%)	*p*	No *n* (%)	Yes *n* (%)	*p*
**Observations**	613 (100)	571 (93.1)	42 (6.9)		566 (92.3)	47 (7.7)		592 (96.6)	21 (3.4)	
**Mean ± SD**		2.46 ± 4.434			2.12 ± 3.655			3.19 ± 4.726		
**Gender**										
Male	165 (26.9)	147 (25.7)	18 (42.9)	0.016 ^a^	153 (27.0)	12 (25.5)	0.866 ^a^	161 (27.2)	4 (19.0)	0.616 ^b^
Female	448 (73.1)	424 (74.3)	24 (57.1)		413 (73.0)	35 (74.5)		431 (72.8)	17 (81.0)	
**Age**										
60–65	342 (55.8)	325 (56.9)	17 (40.5)	0.038 ^a^	319 (56.4)	23 (48.9)	0.325 ^a^	327 (55.2)	15 (71.4)	0.181 ^a^
66–	271 (44.2)	246 (43.1)	25 (59.5)		247 (43.6)	24 (51.1)		265 (44.8)	6 (28.6)	
**Marital status**										
Married	539 (87.9.)	505 (88.4)	34 (81.0)	0.215 ^a^	503 (88.9)	36 (76.6)	0.013 ^a^	522 (88.2)	17 (81.0)	0.305 ^b^
Single	74 (12.1)	66 (11.6)	8 (19.0)		63 (11.1)	11 (23.4)		70 (11.8)	4 (19.0)	
**Education level**										
Below upper-secondary	504 (82.2)	472 (82.7)	32 (76.2)	0.536 ^a^	464 (82.0)	40 (85.1)	0.855 ^a^	490 (82.8)	14 (66.7)	0.162 ^a^
upper secondary	91 (14.8)	83 (14.5)	8 (19.0)		85 (15.0)	6 (12.8)		85 (14.4)	6 (28.6)	
tertiary education	18 (2.9)	16 (2.8)	2 (4.8)		17 (3.0)	1 (2.1)		17 (2.9)	1 (4.8)	
**Monthly income**										
CNY 0–100	210 (34.3)	194 (34.0)	16 (38.1)	0.579 ^b^	190 (33.6)	20 (42.6)	0.549 ^a^	203 (34.3)	7 (33.3)	0.860 ^b^
CNY 101–1000	234 (38.2)	216 (37.8)	18 (42.9)		216 (38.2)	18 (38.3)		225 (38.0)	9 (42.9)	
CNY 1001–2000	67 (10.9)	65 (11.4)	2 (4.8)		63 (11.1)	4 (8.5)		66 (11.1)	1 (4.8)	
CNY ≥ 2001	102 (16.6)	96 (16.8)	6 (14.3)		97 (17.1)	5 (10.6)		98 (16.6)	4 (19.0)	
**Employment**										
Employed	53 (8.6)	46 (8.0)	7 (16.7)	0.040 ^a^	48 (8.5)	5 (10.6)	0.861 ^a^	48 (8.1)	5 (23.8)	0.018 ^a^
Retired and having income	126 (20.6)	114 (20.0)	12 (28.6)		116 (20.5)	10 (21.3)		120 (20.3)	6 (28.6)	
Unemployed and having no income	434 (70.8)	411 (72.0)	23 (54.8)		402 (71.0)	32 (68.1)		424 (71.6)	10 (47.6)	
**Hukou**										
Rural	525 (85.6)	488 (85.5)	37 (88.1)	0.639 ^a^	483 (85.3)	42 (89.4)	0.449 ^a^	508 (85.8)	17 (81.0)	0.533 ^b^
Urban	88 (14.4)	83 (14.5)	5 (11.9)		83 (14.7)	5 (10.6)		84 (14.2)	4 (19.0)	
**Migration reason**										
Taking care of grandchildren	533 (86.9)	500 (87.6)	33 (78.6)	0.028 ^b^	496 (87.6)	37 (78.7)	0.01 ^a^	518 (87.5)	15 (71.4)	0.016 ^b^
Curing a disease or rehabilitation	18 (2.9)	14 (2.4)	4 (9.5)		13 (2.3)	5 (10.6)		15 (2.5)	3 (14.3)	
Others	62 (10.1)	57 (10.0)	5 (11.9)		57 (10.1	5 (10.6)		59 (10.0)	3 (14.3)	
**Migration range**										
Trans-county	430 (70.1)	404 (70.8)	26 (61.9)	0.285 ^b^	407 (71.9)	23 (48.9)	0.002 ^b^	417 (70.4)	13 (61.9)	0.503 ^b^
Trans-city	130 (21.2)	117 (20.5)	13 (31.0)		110 (19.4)	20 (42.6)		125 (21.1)	5 (23.8)	
Trans-province	53 (8.6)	50 (8.8)	3 (7.1)		49 (8.7)	4 (8.5)		50 (8.4)	3 (14.3)	
**Hesitant to migrate**										
Very hesitant	56 (9.1)	50 (8.8)	6 (14.3)	0.443 ^b^	51 (9.0)	5 (10.6)	0.294 ^a^	51 (8.6)	5 (23.8)	0.019 ^b^
A little hesitant	45 (7.3)	42 (7.4)	3 (7.1)		39 (6.9)	6 (12.8)		42 (7.1)	3 (14.3)	
Not at all hesitant	512 (83.5)	479 (83.9)	33 (78.6)		476 (84.1)	36 (76.6)		499 (84.3)	13 (61.9)	
**Chronic diseases**										
No	351 (57.3)	337 (59.0)	14 (33.3)	0.001 ^a^	334 (59.0)	17 (36.2)	0.002 ^a^	345 (58.3)	6 (28.6)	0.007 ^a^
**Yes**	262 (42.7)	234 (41.0)	28 (66.7)		232 (41.0)	30 (63.8)		247 (41.7)	15 (71.4)	
**Health Status**										
Good	455 (74.2)	436 (95.8)	135 (85.4)	<0.001 ^a^	436 (77.0)	19 (40.4)	<0.001 ^a^	447 (75.5)	8 (38.1)	<0.001 ^a^
Moderate or poor	158 (25.8)	19 (4.2)	42 (25.8)		130 (23.0)	28 (59.6)		145 (24.5)	13 (61.9)	
**Outpatient service experience**										
No	445 (72.6)	420 (73.6)	25 (59.5)	0.049 ^a^	421 (74.4)	24 (51.1)	0.001 ^a^	435 (73.5)	10 (47.6)	0.013 ^a^
Yes	168 (27.4)	151 (26.4)	17 (40.5)		145 (25.6)	23 (48.9)		157 (26.5)	11 (52.4)	
**Inpatient service experience**										
No	509 (83.0)	488 (85.5)	21 (50.0)	<0.001 ^a^	487 (86.0)	22 (46.8)	<0.001 ^a^	497 (84.0)	12 (57.1)	0.001 ^a^
Yes	104 (17.0)	83 (14.5)	21 (50.0)		79 (14.0)	25 (53.2)		95 (16.0)	9 (12.9)	
**Going to hospital alone**										
No	331 (54.0)	315 (55.2)	16 (38.1)	0.039 ^a^	315 (55.7)	16 (34.0)	0.004 ^a^	326 (55.1)	5 (23.8)	0.005 ^a^
Yes	282 (46.0)	256 (44.8)	26 (61.9)		251 (44.3)	31 (66.0)		266 (44.9)	16 (76.2)	
**Buying medicine alone**										
No	275 (44.9)	261 (45.7)	14 (33.3)	0.148 ^a^	259 (45.8)	16 (34.0)	0.129 ^a^	269 (45.4)	6 (28.6)	0.810 ^a^
Yes	338 (55.1)	310 (54.3)	28 (66.7)		307 (54.2)	31 (66.0)		323 (54.6)	15 (71.4)	
**Financial stress on medical costs**										
No	233 (38.0)	227 (39.8)	6 (14.3)	<0.001 ^a^	227 (40.1)	6 (12.8)	<0.001 ^a^	232 (39.2)	1 (4.8)	<0.001 ^b^
Moderate	254 (41.4)	238 (41.7)	16 (38.1)		238 (42.0)	16 (34.0)		250 (42.2)	4 (19.0)	
Yes	126 (20.6)	106 (18.6)	20 (47.6)		101 (17.8)	25 (53.2)		110 (18.6))	16 (76.2)	
**Relationship with neighbors**										
Bad	91 (14.8)	80 (14.0)	11 (26.2)	0.001 ^a^	88 (15.5)	3 (6.4)	0.006 ^b^	87 (14.7)	4 (19.0)	0.077 ^b^
fairly bad	86 (14.0)	76 (13.3)	10 (23.8)		77 (13.6)	9 (19.1)		82 (13.9)	4 (19.0)	
fairly good	138 (22.5)	125 (21.9)	13 (31.0)		119 (21.0)	19 (40.4)		130 (22.0)	8 (38.1)	
Good	298 (48.6)	290 (50.8)	8 (19.0)		282 (49.8)	16 (34.0)		293 (49.5)	5 (23.8)	
**Relationship with friends**										
Bad	91 (14.8)	81 (14.2)	10 (23.8)	0.002 ^a^	86 (15.2)	5 (10.6)	0.008 ^a^	87 (14.7)	4 (19.0)	0.180 ^b^
fairly bad	93 (15.2)	80 (14.0)	13 (31.0)		80 (14.1)	13 (27.7)		90 (15.2)	3 (14.3)	
fairly good	134 (21.9)	125 (21.9)	9 (21.4)		119 (21.0)	15 (31.9)		126 (21.3)	8 (38.1)	
Good	295 (48.1)	285 (49.9)	10 (23.8)		281 (49.6)	14 (29.8)		289 (48.8)	6 (28.6)	
**Couple support**										
No	100 (16.3)	91 (15.9)	9 (21.4)	0.353 ^a^	86 (15.2)	14 (29.8)	0.009 ^a^	94 (15.9)	6 (28.6)	0.122 ^a^
Yes	513 (83.7)	480 (84.1)	33 (78.6)		480 (84.8)	33 (70.2)		498 (84.1)	15 (71.4)	
**Children support**										
No	36 (5.9)	26 (4.6)	10 (23.8)	<0.001 ^a^	26 (4.6)	10 (21.3)	<0.001 ^a^	27 (4.6)	9 (42.9)	<0.001 ^a^
Yes	577 (94.1)	545 (95.4)	32 (76.2)		540 (95.4)	37 (78.7)		565 (95.4)	12 (57.1)	
**Sibling support**										
No	297 (48.5)	270 (47.3)	27 (64.3)	0.038 ^a^	266 (47.0)	31 (66.0)	0.012 ^a^	285 (48.1)	12 (57.1)	0.507 ^a^
Yes	316 (51.5)	301 (52.7)	15 (35.7)		300 (53.0)	16 (34.0)		307 (51.9)	9 (42.9)	
**Other members support**										
No	311 (50.7)	282 (49.4)	29 (69.0)	0.014 ^a^	282 (49.8)	29 (61.7)	0.118 ^a^	299 (50.5)	12 (57.1)	0.55 ^a^
Yes	302 (49.3)	289 (51.6)	13 (31.0)		284 (50.2)	18 (38.3)		293 (49.5)	9 (42.9)	
**Economic support when in trouble**										
No	15 (2.4)	11 (1.9)	4 (19.0)	0.015 ^b^	13 (2.3)	2 (4.3)	0.322 ^b^	11 (1.9)	4 (19.0)	0.001 ^b^
Yes	598 (97.6)	560 (98.1)	38 (81.0)		553 (97.7)	45 (95.7)		560 (98.1)	38 (81.0)	
**Comfort support when in trouble**										
No	6 (1.0)	3 (0.5)	3 (7.1)	0.005 ^b^	5 (0.9)	1 (2.1)	0.382 ^b^	5 (0.8)	1 (4.8)	0.189 ^b^
Yes	607 (99.0)	568 (99.5)	39 (92.9)		561 (99.1)	46 (97.9)		587 (99.2)	20 (95.2)	

Note: SD= standardized deviation; ^a^ = Chi-square test, ^b^ = Fisher exact test.

**Table 2:** Binary logistic regression for relationships between related variables and depression of MEFC.

Variables	Model 1 Socio-Demographic Variables		Model 2 Model 1+ Health Services Utilization		Model 3 Model 2 + Social Support	
	OR	95% CI	OR	95% CI	OR	95% CI
**Gender**						
Male	1.0		1.0		1.0	
Female	0.605	0.303, 1.207	0.576	0.270, 1.227	0.702	0.296, 1.665
**Age**						
60–65	1.0		1.0		1.0	
66–	1.960	0.957, 4.010	1.739	0.817, 3.702	1.720	0.746, 3.968
**Employment**						
Employed	1.0		1.0		1.0	
Retired and having income	0.447	0.153, 1.304	0.457	0.144, 1.449	0.618	0.161, 2.365
Unemployed and having no income	0.307 *	0.113, 0.833	0.211 **	0.069, 0.645	0.262 *	0.070, 0.979
**Migration reason**						
Taking care of grandchildren	1.0		1.0		1.0	
Curing a disease or rehabilitation	3.425 *	1.030, 11.392	2.312	0.611, 8.748	2.710	0.574, 12.805
Others	0.853	0.308, 2.634	0.679	0.223, 2.2065	2.419	0.200, 2.319
**Chronic diseases**						
No			1.0		1.0	
Yes			1.150	0.531, 2.2489	1.394	0.594, 3.273
**Health Status**						
Good					1.0	
Moderate and poor			1.773	0.810, 3.878	1.639	0.691, 3.888
**Outpatient service experience**						
No			1.0		1.0	
Yes			0.839	0.391, 1.800	0.650	0.277, 1.528
**Inpatient service experience**						
No			1.0		1.0	
Yes			3.496 **	1.566, 7.808	2.068	0.847, 5.049
**Go to hospital alone**						
No			1.0		1.0	
Yes			1.307	0.629, 2.712	1.361	0.591, 3.133
**Pressure on medical costs**						
No			1.0		1.0	
Moderate			1.668	0.591, 4.709	3.454 *	1.010, 11.809
Yes			3.942 **	1.266, 12.272	6.557 **	1.717, 25.044
**Relationship with neighbors**						
Bad					1.0	
fairly bad					0.963	0.233, 3.978
fairly good					0.807	0.184, 3.545
Good					0.250	0.047, 1.328
**Relationship with friends**						
Bad					1.0	
fairly bad					1.798	0.410, 7.890
fairly good					1.081	0.208, 5.620
Good					1.465	0.275, 7.814
**Children support**						
No					1.0	
Yes					0.257 *	0.085, 0.780
**Sibling support**						
No					1.0	
Yes					1.081	0.208, 5.620
**Other members support**						
No					1.0	
Yes					0.595	0.235, 1.058
**Economic support when in trouble**						
No					1.0	
Yes					0.495	0.068, 3.593
**Comfort support when in trouble**						
No					1.0	
Yes					0.018 **	0.001, 0.245

Note: * *p* < 0.05; ** *p* < 0.01; OR = odds ratio; CI = confidence interval.

**Table 3:** Binary logistic regression for relationships between related variables and anxiety of IMEFC.

Variables	Model 1 Socio-Demographic Variables		Model 2 Model 1+ Health Services Utilization		Model 3 Model 2 + Informal Social Support	
	OR	95% CI	OR	95% CI	OR	95% CI
**Marital status**						
Married	1.0		1.0		1.0	
Single	1.920	0.863, 4.269	1.538	0.647, 3.657	1.798	0.418, 7.724
**Migration reason**						
Taking care of grandchildren	1.0		1.0		1.0	
Curing a disease or rehabilitation	3.837 *	1.163, 12.657	3.098	0.817, 11.744	2.850	0.679, 11.957
Others	0.973	0.349, 2.714	0.979	0.0320, 2.997	1.015	0.320, 3.218
**Migration range**						
Trans-county	1.0		1.0		1.0	
Trans-city	3.004 **	1.576, 5.725	2.959 *	1.457, 6.008	3.198 **	1.462, 6.999
Trans-province or country	1.111	0.349, 3.541	1.150	0.298, 4.428	0.97	0.234, 4.032
**Chronic diseases**						
No	1.0		1.0		1.0	
Yes			1.071	0.507, 2.262	0.915	0.413, 2.030
**Health Status**						
Good			1.0		1.0	
Moderate or poor			1.816	0.857, 3.849	2.179	0.971, 4.890
**Outpatient service experience**						
No			1.0		1.0	
Yes			1.277	0.620, 2.2631	1.207	0.556, 2.619
**Inpatient service experience**						
No			1.0		1.0	
Yes			3.584 **	1.709, 7.515	3.818 **	1.687, 8.644
**Go to hospital alone**						
No			1.0		1.0	
Yes			1.651	0.799, 3.413	1.121	0.510, 2.462
**Financial stress on medical**						
No			1.0		1.0	
Moderate			1.310	0.460, 3.728	1.600	0.537, 4.763
Yes			3.014 *	1.017, 8.929	3.726 *	1.174, 11.819
**Relationship with neighbors**						
Bad					1.0	
fairly bad					2.017	0.329, 12.368
fairly good					4.458	0.697, 2.8.531
Good					5.318	0.762, 37.121
**Relationship with friends**						
Bad					1.0	
fairly bad					2.876	0.565, 14.646
fairly good					2.469	0.416, 14.662
Good					0.710	0.108, 4.687
**Couple support**						
No					1.0	
Yes					1.060	0.284, 3.959
**Children support**						
No					1.0	
Yes					0.198 *	0.066, 0.598
**Sibling support**						
No					1.0	
Yes					0.607	0.274, 1.342

Note: * *p* < 0.05; ** *p* < 0.01; OR = odds ratio; CI = confidence interval.

**Table 4:** Binary logistic regression for relationships between related variables and stress of IMEFC.

Variables	Model 1 Socio-Demographic Variables			Model 2 Model 1+ Health Services Utilization	Model 3 Model 2 + Informal Social Support	
	OR	95% CI	OR	95% CI	OR	95% CI
**Employment**						
Employed	1.0		1.0		1.0	
Retired and having income	0.375	0.103, 1.369	0.477 **	0.109, 2.077	1.061	0.154, 7.313
Unemployed	0.175 **	0.053, 0.585	0.116	0.028, 0.475	0.224	0.034, 1.463
**Migration reason**						
Taking care of grandchildren	1.0		1.0		1.0	
Curing a disease or rehabilitation	7.499 **	1.808, 31.110	6.192	1.133, 33.838	12.702 *	1.681, 95.968
Others	1.496	0.393, 6.696	1.219 *	0.269, 5.512	2.582	0.380, 17.560
**Hesitant to migrate**						
Very hesitant	1.0		1.0		1.0	
A little hesitant	0.787	0.167, 3.707	0.807	0.140, 4.637	0.897	0.086, 9.325
Not at all hesitant	0.203 **	0.065, 0.634	0.275 *	0.078, 0.975	0.370	0.066, 2.081
**Chronic diseases**					0.754	0.177, 3.202
No			1.0		1.0	
Yes			1.319	0.424, 4.107	1.189	0.294, 4.817
**Health Status**						
Good			1.0		1.0	
Moderate and poor			1.309	0.407, 4.207	2.275	0.518, 9.989
**Outpatient service experience**						
No			1.0		1.0	
Yes			1.268	0.437, 3.675	0.754	0.177, 3.202
**Inpatient service experience**						
No			1.0		1.0	
Yes			1.465	0.463, 4.639	1.089	0.305, 3.886
**Go to hospital alone**						
No			1.0		1.0	
Yes			2.334	0.700, 7.779	1.963	0.388, 9.920
**Financial stress on medical**						
No			1.0		1.0	
Moderate			2.171	0.217, 21.702	3.638	0.274, 48.292
Yes			18.291 *	1.944, 172.101	32.155 *	2.321, 445.393
**Children support**						
No					1.0	
Yes					0.055 *	0.010, 0.301
**Economic support when in trouble**						
No					1.0	
Yes					0.012 *	0.001, 0.136

Note: * *p* < 0.05; ** *p* < 0.01; OR = odds ratio; CI= confidence interval.

## Data Availability

The data that support the findings of this study are available from the corresponding author, upon request.
